# Characterizing Twitter Influencers in Radiation Oncology

**DOI:** 10.1016/j.adro.2022.100919

**Published:** 2022-03-23

**Authors:** Luca F. Valle, Fang-I Chu, Marc Smith, Chenyang Wang, Percy Lee, Drew Moghanaki, Fumiko L. Chino, Michael L. Steinberg, Ann C. Raldow

**Affiliations:** a UCLA Department of Radiation Oncology, University of California Los Angeles, Los Angeles, California; b Social Media Research Foundation, Redwood City, California; c Department of Radiation Oncology, MD Anderson Cancer Center, Houston, Texas; d Department of Radiation Oncology, Memorial Sloan Kettering Cancer Center, New York, New York

## Abstract

**Purpose:**

Both the superstructures of virtual discourse in radiation oncology and the entities occupying influential positions in the social media landscape of radiation oncology remain poorly characterized.

**Methods and Materials:**

NodeXL Pro was used to prospectively sample all tweets with the hashtag #radonc every 8 to 10 days during the course of 1 year (December 4, 2018, to November 29, 2019). Twitter handles were grouped into conversational clusters using the Clauset-Newman-Moore community detection algorithm. For each sample period, the top 10 #radonc Twitter influencers, defined using betweenness centrality, were categorized. Influencers were scored in each sample period according to their top 10 influence rank and summarized with descriptive statistics. Linear regression assessed for characteristics that predicted higher influence scores among top influencers.

**Results:**

In the study, 684,000 tweets were sampled over 38 periods. #radonc tweets took on the crowd superstructure of a hub-and-spoke broadcast network formed when prominent individuals are widely repeated by many audience members. Professional societies were the most influential category of Twitter handles with an average influence score of 7.63 out of 10 (standard deviation [SD] = 1.94). When industry handles were present among top 10 influencers, they exhibited the second highest average influence scores (6.75, SD = 1.06), followed by individuals with scores of 5.28 (SD = 0.43). The categories of influencers were stable during the course of 1 year. The role of attending physician, radiation oncology specialty, male sex, academic practice, and US-based handles in North America were predictors of higher influence score.

**Conclusions:**

Twitter influencers in radiation oncology represent a diverse group of people and organizations, but male academic radiation oncologists based in North America occupy particularly influential positions in virtual communities broadly characterized as “hub and spoke” broadcast networks. Periodic network-based analyses of the social media discourse in radiation oncology are warranted to maintain an awareness of the handles that are influencing discussions on Twitter and ensure that social media utilization continues to contribute to the field of radiation oncology in a meaningful way.

## Introduction

Social media is increasingly being used by both radiation oncologists^1^ and patients^2^ and the potential benefits of social media engagement within the oncology community are myriad. If used responsibly,^3^ Twitter in particular has the potential to become a space for multidisciplinary exchanges where patients, health professionals, and researchers can interact and share thoughts and concepts more equitably. Recently, Twitter has served as an amplifying voice to public health messages,^4^ a tool for effectively assessing perceptions regarding vaccine-based cancer prevention,^5^ a forum for journal clubs,^6^ and has even been suggested as a strategy for bolstering clinical trial enrollment.^7^ There has also been interest in its use for reinforcing the critical role radiation therapy plays in oncologic care with messaging that reaches and educates patients more directly.^8^

As a consequence of these expanding uses of social media, vast networks of discourse have emerged on Twitter, where influence is exerted over opinions and potentially clinical decision-making. However, all voices are not equally influential on Twitter, and despite significant increases in Twitter content, very little is known about the people and institutions that occupy influential positions in the rapidly expanding social media landscape of radiation oncology. We sought to use network analysis to characterize the most influential Twitter handles using the hashtag #radonc during the course of a year.

## Methods

### Twitter sampling

NodeXL Pro software (Social Media Research Foundation, Redwood City, CA) was created to support open scholarship related to social media and to generate and host open data. We used this software to prospectively sample tweets with the hashtag #radonc over the course of one year (December 4, 2018 to November 29, 2019). 18,000 tweets are captured per sampling, resulting in an 8- to 10-day lookback period for each NodeXL Pro sampling. Twitter was thus sampled every 8 to 10 days to minimize both gaps and overlap in Twitter activity. This resulted in a total of 38 sampling periods during the course of the year. Institutional Review Board approval was waived given that all information analyzed was publicly available.

### Twitter network crowd depiction

NodeXL Pro was used to graphically visualize conversational superstructures with the hashtag #radonc for each period sampled. Twitter handles were grouped into conversational clusters using the Clauset-Newman-Moore community detection algorithm, as previously described.^9^

### Identification of influential Twitter handles

Influencers were defined using network measures of “betweenness centrality,” which is a validated^10^ approach for identifying key handles in critical locations that create bridges between conversational clusters. Because betweenness centrality depends on being broadly connected across cluster and group boundaries, this reflects the significant role these handles play in Twitter discussions.^11^ Influence should be distinguished from popularity, which assesses “in degree” connections to a target handle (ie, replies, retweets, or mentions), and was not considered in this analysis. Follower count was also not considered in this analysis because it does not serve as a strong proxy for engagement, awareness, or interaction.

**Figure 1:**
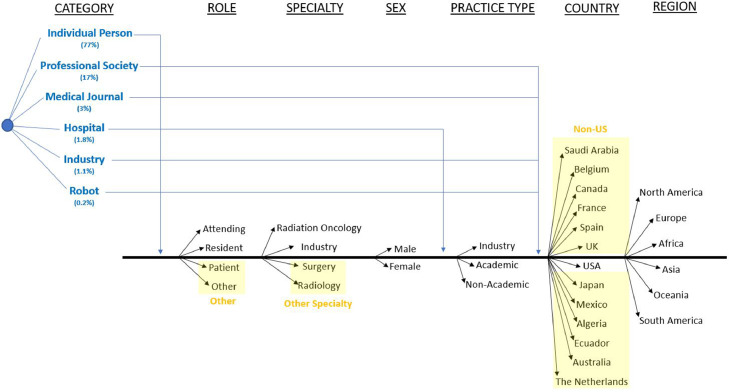
Twitter influencer categorization taxonomy. Categorization schema for each top influential Twitter user. The 6 categories of influencers depicted in blue were subcategorized into the categories listed in black. Grouped categories of “other,” “other specialty,” and “non-US” are highlighted in yellow shading.

For each sample period, the top 10 Twitter influencers using the hashtag #radonc were identified using betweenness centrality and categorized according to the schema in Fig. 1. Handles were first categorized as an individual person, a professional society, a medical journal, a hospital, industry, or a robot (nonhuman controlled account). Individuals were further categorized by role (attending, resident, or “other” [including patients]), specialty (radiation oncology, industry, or “other specialty” [including surgery and radiology], sex (male, female), practice type (industry, academic, or nonacademic), country of practice (US or non-US), and region of practice (North America, Europe, Africa, Asia, Oceania, or South America). Hospitals were categorized according to practice type, country, and region, whereas professional societies, medical journals, industry, and robots were categorized by just country and region.

Data used for characterization were obtained from the Twitter user's profile and online searches. Handles were considered academic if they had an academic appointment with a title of professor, associate professor, or assistant professor. If title was not listed on the website, they were assumed to be academic if they worked at a teaching hospital or an academic center. Clinical oncologists in the United Kingdom were considered radiation oncologists for the purpose of this analysis. All resident physician handles were categorized as academic. Sex was determined by self-identification on Twitter, on academic profiles, and in cases of ambiguity, using an algorithmic assessment of sex probabilities based on first name.^12^

### Quantitative analysis of Twitter influence over time

For each period sampled, influence was scored on a 10-point scale according to handle rank in the top 10 list (a rank score of 10 for the number one influencer, a rank score of 9 points for the number 2 influencer, etc). Rank scores for each period of the study year were grouped according to the previously established categories and summarized using descriptive statistics. Only categories present in a given period were included in the influence score calculation for that period. Scores were also used to generate stacked barplots, which summarize the proportion of influence scores in each of the 38 periods. Descriptive statistics also summarized the Twitter handles with the highest influence scores during the entire study year.

Finally, linear regression analysis was applied to evaluate the relationship between influence score as a response variable and independent variables including period and categorical influencer characteristics. Likelihood ratio test was conducted to assess the overall significance of categorical variables with 3 or more levels. The significance level was set at 5%. All statistical analyses were carried out using R 3.6.0.^13^

## Results

**Figure 2:**
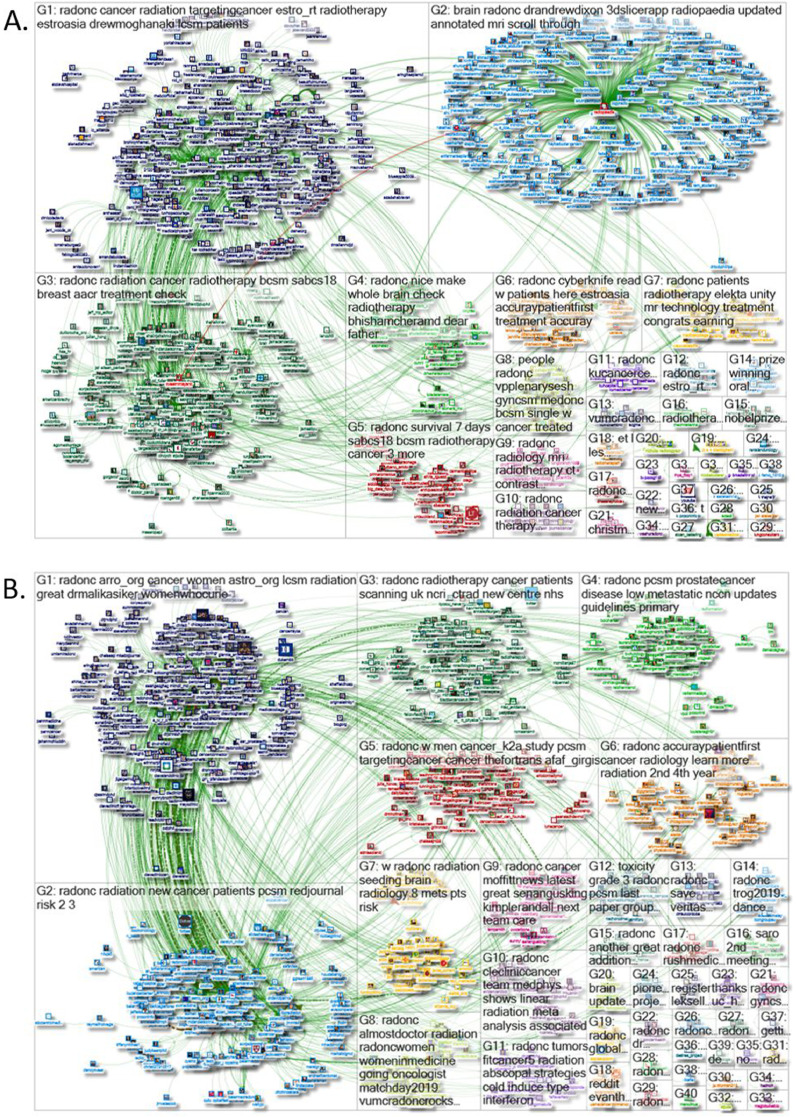

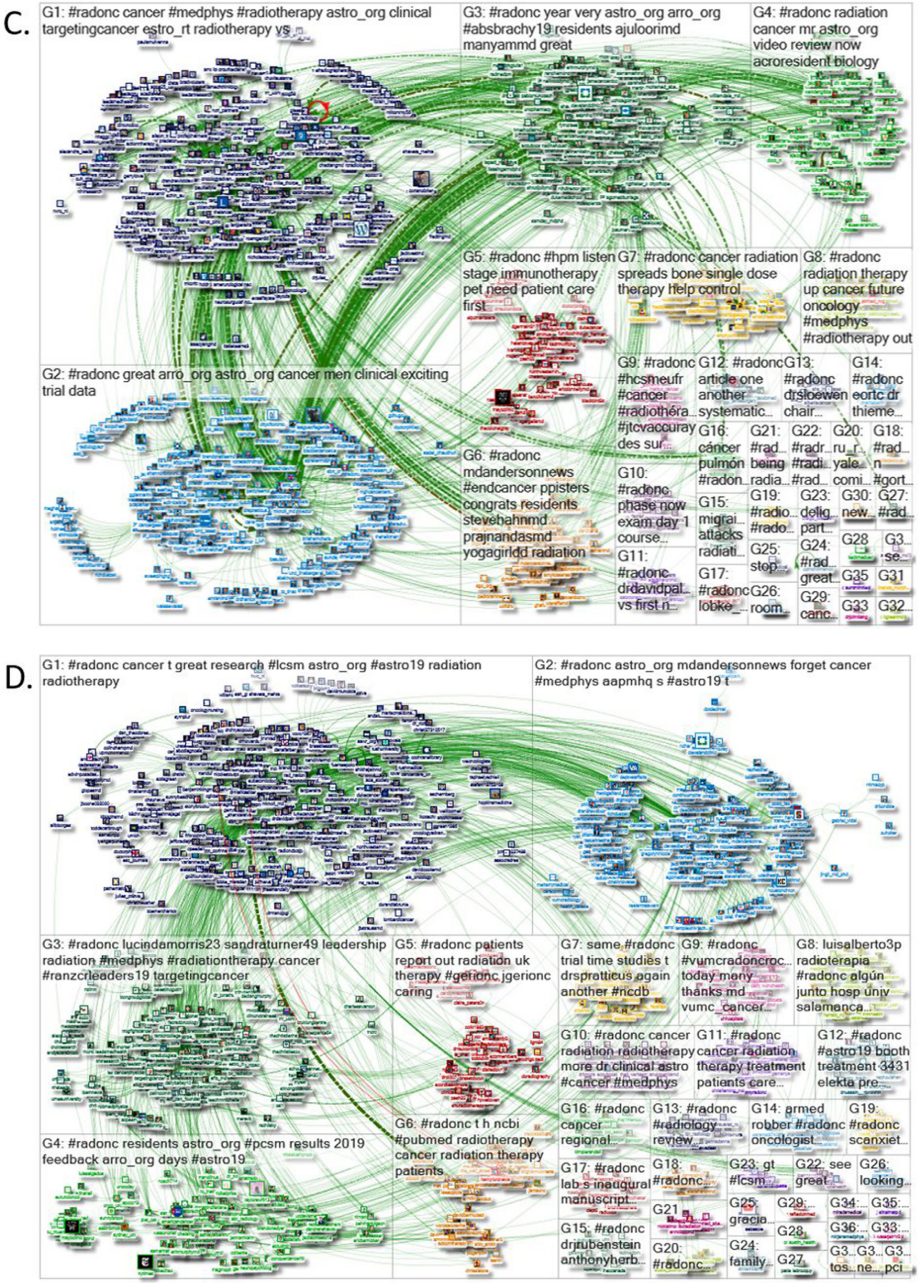
Network crowd diagrams. Network crowd diagrams depicting the superstructure of social media relationships among Twitter handles exchanging tweets with the hashtag #radonc from 4 representative periods sampled on (A) December 11, 2018, (B) March 13, 2019, (C) June 23, 2019, and (D) September 6, 2019. Each Twitter user is represented by their profile picture, and size of the picture correlates with their number of Twitter followers at the time of sampling. Twitter handles are color-coded and organized into clusters according to the conversational hashtags that unite everyone in that group. Conversation groups are loosely contained within boxes labeled G1, G2, G3 and organized in descending order according number of handles in the group. Green lines represent links between 2 Twitter handles who follow, reply to, or mention one another. Circles represent tweets that do not mention or reply to another Twitter handle.

A total of 684,000 tweets were considered for analysis. Network crowd diagrams from four periods sampled during the study year are shown in Fig. 2. Using the taxonomy formalized by the Social Media Research Foundation,^11^ there are six possible conversational archetypes that characterize social media conversations: polarized crowd, tight crowd, brand clusters, community clusters, broadcast network, and support network. In tweets tagged with #radonc, the network crowd superstructure has the predominant features of a broadcast network, with elements of community clusters and a tight crowd evident as well.

A broadcast network is characterized by Twitter commentary around the publishing of new information, creating a distinctive “hub and spoke” structure exemplified in panels A and D of Fig. 2, where many handles share information that was tweeted out by a few prominent news sources. This “hub and spoke” structure is characterized by a largely unpolarized crowd of discussants with a few trusted experts at the center of the hub who serve as information distributors and those interacting with that information flanking them at the periphery of the spokes. Moreover, the members of this broadcast network audience are often connected only to the hub news source, without connecting to one another, though there can be smaller subgroups of densely connected people who discuss new developments with one another. There are also more subtle features of tight crowds, particularly in Fig. 2 C, where discussions are characterized by highly interconnected people with fewer isolated participants. Conferences and professional discussions tend to take on this form, which is an important example of how networked learning communities function to share information and provide mutual support. In community clusters, popular Twitter topics may develop multiple smaller groups, which often form around a few hubs each with its own audience, influencers, and sources of information. These communities are likened to bazaars that host multiple centers of activity and can create medium to large sized groups in addition to a fair number of smaller isolated groups, as shown in panels B and C of Fig. 2. There are no major qualitative structural changes to the conversational superstructure observed throughout the study year.

**Figure 3:**
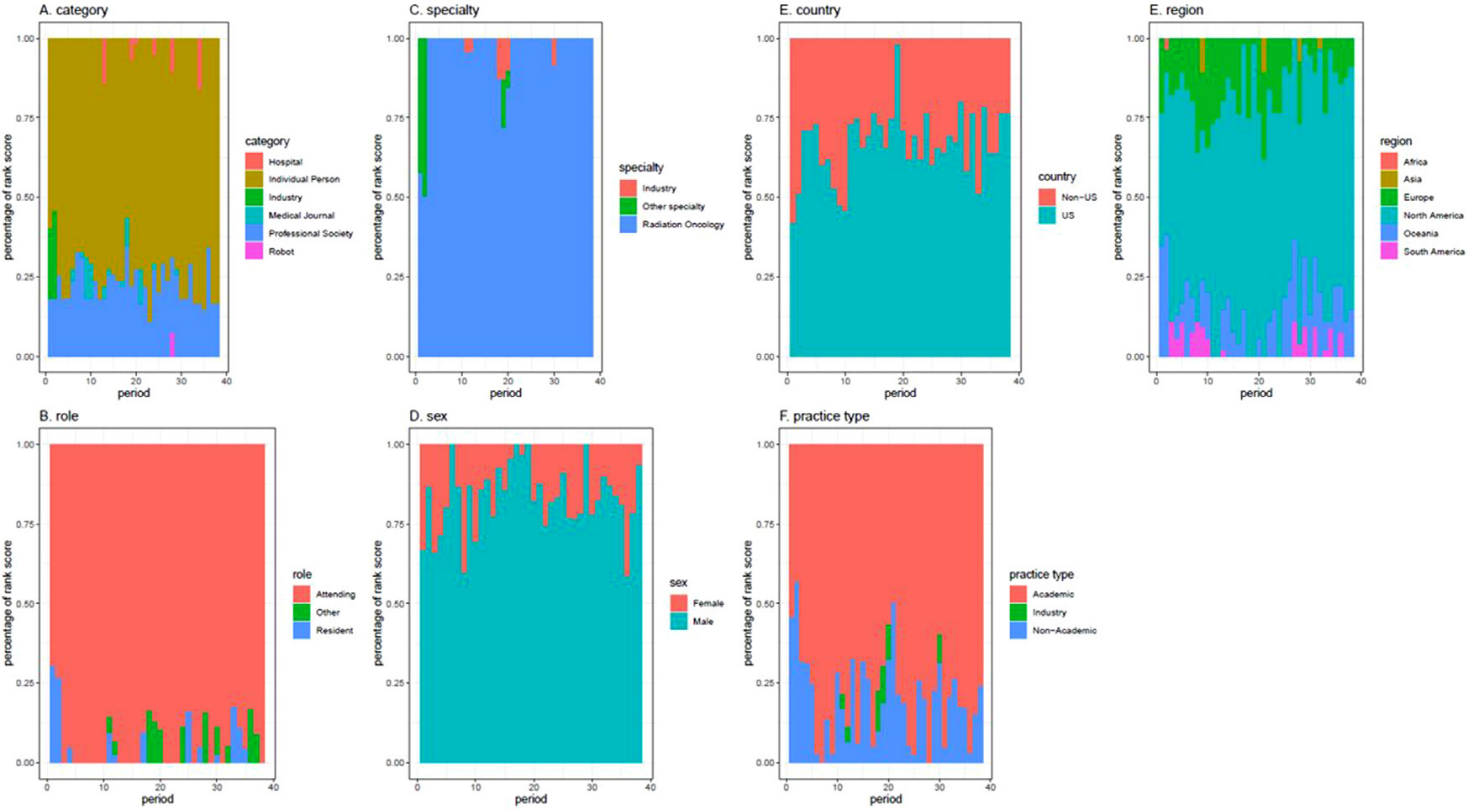
Proportional changes in categorical influence scores over 1 year. Stacked bar plots showing the relative proportions of influencer scores (y-axis) for each of the 38 periods sampled (x-axis) across (A) the categories of individual person, hospital, industry, medical journal, professional society, and robot. Changes in influencer characteristics within the subcategories of (B) role, (C) specialty, (D) sex, (E) country, (F) practice type, and (E) region are also shown.

Figure 3 demonstrates how the proportions of influential handles changed during the study year. There appears to be relative stability in the proportion of influencer categories over time. Individual people and professional societies were most often the most influential handles. Among subcategories, the handles of attending physicians, radiation oncologists, men, North American-based Tweeters, US-based Tweeters, and academics were most frequently included in the list of top 10 influential accounts.

For periods where a given influencer category was present in the top 10 list, descriptive statistics of average influence scores in each category are presented in Table 1. During the 38 periods sampled, professional societies (including American Society for Radiation Oncology [ASTRO], Association of Residents in Radiation Oncology [ARRO], and European Society Radiation Oncology [ESTRO]) were the most influential category of Twitter handles, with an average influence score of 7.63 out of 10 (standard deviation [SD] = 1.94). When industry handles were present among top 10 influencers, their importance was notable, with the second highest average aggregate influence score (6.75, SD = 1.06). This was followed by individuals, who had an average aggregate score of 5.28 (SD = 0.43). When attending physicians were top influencers, their average aggregate influence score was 5.43 (SD = 0.60) compared with a score of 4.00 for resident physicians (SD = 2.56). Influence scores were similar for radiation oncology handles compared with handles from other specialties. Top influencers who were men tended to have higher influence scores than top women influencers, with average aggregate scores of 5.46 and 4.66, respectively. Finally, top influential handles classified as academic, North American-based, and more specifically US-based, all tended to have greater influence than their categorical counterparts.

**Table 1:** Average influence score according to category of influencer

	Average influence score	Standard deviation
Category		
Individual person (unique handles = 49)	5.28	0.43
Robot (unique handles = 1)	4.00	NA
Hospital (unique handles = 4)	4.42	2.42
Industry (unique handles = 2)	6.75	1.06
Medical journal (unique handles = 7)	2.73	2.24
Professional Society (unique handles = 9)	7.63	1.94
Role		
Attending Physician (unique handles = 39)	5.43	0.60
Resident physician (unique handles = 6)	4.00	2.56
Other (unique handles = 4)	3.68	1.38
Specialty		
Radiation Oncology (unique handles = 45)	5.29	0.63
Other specialty (unique handles = 3)	5.63	2.50
Industry (unique handles = 1)	3.50	1.22
Sex		
Women (unique handles = 8)	4.66	1.73
Men (unique handles = 41)	5.46	0.65
Country		
US (unique handles = 35)	6.16	0.69
Non-US (unique handles = 37)	4.48	0.98
Practice		
Academic (unique handles = 38)	5.51	0.70
Nonacademic (unique handles = 11)	4.72	2.32
Industry (unique handles = 1)	3.50	1.22
Region		
North America (unique handles = 37)	6.10	0.65
Africa (unique handles = 1)	2.00	NA
Asia (unique handles = 4)	4.50	1.91
Europe (unique handles = 19)	4.00	1.58
Oceania (unique handles = 9)	5.60	1.84
South America (unique handles = 2)	4.13	1.77

*Abbreviation:* NA, not applicable.

When the scores from top Twitter influences from each period were considered in aggregate, an anonymized list of top Twitter handles for the entire study year is presented in Table 2. The top spot was occupied by ASTRO and the remaining 9 spots were occupied by individuals.

**Table 2:** Top 10 influential Twitter handles using the hashtag #radonc

Handle rank	Total influence rank score	No. of periods ranked as a top 10 influencer (n = 38)	Handle category	Handle role	Handle specialty	Handle sex	Handle country	Handle practice	Handle region
No. 1	351	38	Professional society	NA	NA	NA	US	NA	North America
No. 2	305	35	Individual person	Attending	Radiation oncology	Male	US	Academic	North America
No. 3	204	34	Individual person	Attending	Radiation oncology	Male	US	Academic	North America
No. 4	129	20	Individual person	Attending	Radiation oncology	Male	US	Academic	North America
No. 5	117	23	Individual person	Attending	Radiation oncology	Male	US	Nonacademic	North America
No. 6	91	16	Individual person	Attending	Radiation oncology	Female	Spain	Nonacademic	Europe
No. 7	88	13	Individual person	Attending	Radiation oncology	Male	Australia	Academic	Oceania
No. 8	76	16	Individual person	Attending	Radiation oncology	Female	Australia	Academic	Oceania
No. 9	57	14	Individual person	Attending	Radiation oncology	Female	Mexico	Academic	South America
No. 10	47	10	Individual person	Attending	Radiation oncology	Male	France	Academic	Europe

*Abbreviation:* NA, not applicable.

When considering characteristics of Twitter handles that predict for influence, as shown in Table 3, medical journals had significantly lower influence scores compared with individuals (–2.59, *P* < .001), whereas professional societies had significantly higher scores (2.35, *P* < .001). No differences in influence emerged between individuals, robots, hospitals, and industry (*P* > .2 for all). Resident physicians (–1.43, *P* < .01) and Twitter handles with other roles (–1.79, *P* < .001), including patients and researchers, were both less influential compared with attending physicians. Among handles that could be classified according to specialty, radiation oncologists were similarly influential as those from other specialties but were significantly more influential than industry handles (–1.78, *P* < .001). Male sex predicted for influence (0.8, *P* = .01), albeit with a relatively small difference in average influence score, as did academic practice compared with nonacademic (–0.78, *P* < .05) and industry (–1.99, *P* < .01) groups. With the exception of Oceania, all other regions were home to handles with significantly lower influence scores compared with the North American reference (*P* < .05 for all). Across all categories, influence did not change significantly with time (period *P* > .05 for all). The likelihood ratio test demonstrated the overall significance for the aforementioned categorical variables with 3 or more levels (Table E1).

**Table 3:** Predictors of influential Twitter accounts in radiation oncology

	Estimate	Standard Error	*t* value	*P* Value
Category (reference: individual person)				
(Intercept)	5.44	0.40	13.43	<.001
Robot	–1.22	1.62	–0.75	.45
Hospital	–0.84	0.70	–1.20	.23
Industry	1.33	1.19	1.11	.27
Medical journal	–2.59	0.55	–4.71	<.001
Professional society	2.35	0.37	6.42	<.001
Period	–0.01	0.02	–0.49	.63
Role (reference: attending physician)				
(Intercept)	5.28	0.38	13.92	<.001
Resident	–1.43	0.45	–3.19	<.01
Other	–1.79	0.47	–3.81	<.001
Period	0.01	0.02	0.49	.63
Specialty (reference: radiation oncology)				
(Intercept)	5.21	0.30	17.10	<.001
Other specialty	0.37	0.52	0.72	.48
Industry	–1.78	0.42	–4.21	<.001
Period	0.00	0.01	0.28	.78
Sex (reference: women)				
(Intercept)	4.64	0.35	13.30	<.001
Men	0.80	0.30	2.62	.01
Period	0.00	0.01	0.06	.95
Country (reference: US)				
(Intercept)	6.25	0.22	28.26	<.001
Non-US	–1.69	0.19	–8.66	<.001
Period	0.00	0.01	–0.47	.64
Practice type (reference: academic)				
(Intercept)	5.22	0.44	11.98	<.001
Nonacademic	–0.78	0.39	–2.01	<.05
Industry	–1.99	0.74	–2.70	<.01
Period	0.01	0.02	0.83	.41
Region (reference: North America)				
(Intercept)	6.39	0.33	19.28	<.001
Africa	–4.36	1.50	–2.91	<.01
Asia	–1.56	0.77	–2.02	<.05
Europe	–2.11	0.34	–6.18	<.001
Oceania	–0.48	0.36	–1.33	.19
South America	–1.98	0.45	–4.43	<.001
Period	–0.01	0.01	–1.27	.21

## Discussion

Our findings, driven by network analysis of #radonc Tweets, demonstrate that social media influencers in radiation oncology represent a diverse group of people and institutions from fields not necessarily limited to radiation oncology. They include handles that are managed by industry, hospitals, medical journals, and even robots. Categories of influencers were relatively stable during the study period, rather than dynamic, with academic radiation oncologists comprising a major influential group after ASTRO. Twitter also appears to be an emerging space for resident physicians to influence conversations in radiation oncology. The most consistent influential handle was the ASTRO account.

In an environment where social media discourse may exert a profound effect not only on intellectual exchange in radiation oncology,^1^ but also on the composition and future of the field more broadly,^14^ the need for a comprehensive understanding of the characteristics of influential social media presences in radiation oncology becomes clear. Moreover, analyses of social media networks have the ability to uncover the influence of lesser-known individuals and identify trending topics that drive conversations, engagement, and potentially even behavior. These insights add to what can be learned from surveys, focus groups, or even “sentiment analysis” of tweets,^11^ and offer a data-driven approach to visually disentangling an entirely new forum^15^ for discussion in radiation oncology.

Our analysis of Twitter influencers yielded several novel findings that merit contextualization.

Our report is the first to present data regarding the overall superstructures of social media conversations in radiation oncology. Based on the taxonomy set forth by the Social Media Research Foundation, we uncovered that the predominant conversational landscape has features of a hub-and-spoke broadcast network. This finding confirms what many have suspected about the virtual discourse in radiation oncology: it is characterized by a largely unpolarized crowd of discussants with an educational hub and spoke organization which is well-suited for the dissemination of information from trusted experts within the field. This is in alignment with our finding that academic radiation oncologists in large part comprise the most consistent cohort of #radonc influencers, a finding that also predates the advent of social media. Markedly absent from the conversational superstructure are polarized crowds where little common ground is shared between handles. Although Twitter debates about controversial topics may receive significant attention, it is important to remember that the public discourse about opposing views can be educational, that knowledge-sharing is the dominant #radonc conversational configuration, and that the prevailing patterns of Twitter discoutse suggests a commitment to curing cancer collaboratively, both in the clinic and virtually.

We quantitatively demonstrated that top Twitter influencers are a heterogeneous group of individuals and nonindividuals from varying countries, specialties, practice settings, and levels of training. The relative proportions among categories of influencers did not change significantly over time. Events such as professional meetings,^8^ the publication of new findings, and the gradual increase in social media engagement^1^ do not appear to significantly alter who is influencing the conversation.

Despite the diverse categories of radiation oncology influencers, there are some features that consistently predict for an influential role on Twitter. These include male sex, geographic base in the United States, and academic practice, in line with other work.^1^ This may indicate the wide circulation of a narrow set of viewpoints or opinions on Twitter, and may also highlight an important opportunity to contextualize the content of what is widely shared. Although the gender imbalance in radiation oncology is well-described,^16^^,^^17^ in fields such as health policy and health services research, where representation is more balanced between men and women, women still have been shown to still exhibit less influence on Twitter,^18^ suggesting that representation alone may insufficiently explain our divergent gender-based influence findings.

Although no resident physicians enter into the top 10 influencers overall, individual residents and the Association of Residents in Radiation Oncology handle both made the top 10 influencer list over several sampled periods, suggesting that radiation oncologists in training are wielding notable influence on virtual platforms. Accordingly, social media may offer residents a more accessible avenue for sharing their perspectives, as well as opportunities for direct engagement with patients, providers, policymakers, and other stakeholders in radiation oncology to influence perceptions. The opportunities to influence on social media also likely face fewer barriers compared with day-to-day interactions within academic institutions, where hierarchy is customary. Resident engagement through social media is likely to increase in the future, as a recent survey study demonstrated that residents perceive that social media provides novel educational content and may even help with career development.^19^

Although the content of Tweets was not formally evaluated in this investigation, it is noteworthy when examining the list of top influential handles that over half of those individuals specialize in the management of lung cancer or are active proponents of stereotactic body radiation therapy. The use of stereotactic body radiation therapy in lung cancer, particularly for operable patients, has been an area of debate and may be a reason for the frequency of posts on this topic. This observation indicates that controversial topics may be more likely to engage Twitter users who are interested in radiation oncology. It may also identify an opportunity to broaden the scope of influential conversations on Twitter.

It is interesting that professional societies, such as ASTRO, demonstrate higher influential scores than people. This may be due to ASTRO's role in supporting radiation oncologists across all disease sites. It may also be a result of ASTRO's representation of composite views that may engender greater trust and thus more easily influence conversations on Twitter compared with the views of individuals. Although it may be reassuring to some that a large, professional body occupies the dominant influential footprint on Twitter, other work has shown that even ASTRO's use of Twitter lags behind other professional societies such as American Society of Clinical Oncology and Society of Surgical Oncology,^20^ so this work should not discourage further efforts to bolster meaningful engagement from ASTRO and other radiation oncology constituents on Twitter.

Although other reports have considered Twitter activity in the context of specific campaigns (such as #WomenWhoCurie^21^) or annual meetings,^8^^,^^22^ our report differs in that it provides a comprehensive view of Twitter influence over an entire year. A recent study^22^ by Beroual et al explored a similar topic with notable methodological differences. Although their analysis was limited to people, ours was more comprehensive and evaluated all influential handles (including those belonging to industry, professional societies, and journals) to more accurately characterize all entities potentially influencing virtual discourse. Their analysis also used an arbitrary cutoff of 500 followers to define influencer status, whereas ours harnessed network analysis to incorporate more robust signals of influence such as betweenness centrality.^11^ Other work^1^ describes larger trends in Twitter usage among radiation oncologists while ours provides a more comprehensive characterization of Twitter influencers specifically with the advantage of network analysis of influence.

Although measured activity on Twitter provides a readout of what's being read and discussed, the clinical and professional effect of Twitter influence is uncertain and challenging to quantify. Prior reports have demonstrated no correlation between Twitter influence and H-index, but it may be of interest to assess whether Twitter influence “influences” other career-based metrics including grant acquisition, time to promotion, time to partnership, or time in the search for employment. These studies may be particularly meaningful given recent interest^23^ in adding social media contributions to professional curriculum vitae in medicine. Moreover, while social media may represent an opportunity to cement traditional influence garnered by academic publications^24^ and leadership within specialty societies, Twitter may also offer an alternative pathway for influencing conversations in radiation oncology. The authors are excited by this prospect, as it enables diversification of the voices in our field and helps us move beyond the notion that only those with a specific set of experiences or achievements have a voice.

Twitter influence is likely dynamic in the long-term, and a limitation of this analysis is that it only representes a detailed snapshot over a single year and thus may not reflect future or past influencer patterns in radiation oncology. However, it does hilight future related research opportunities to periodically monitor the social media discourse of our field and maintain a self-awareness of the character and content of our virtual communities, especially given that influencers are occasionally outside of the radiation oncology community. And finally, while efforts to more optimally identify and categorize relevant social media content through hashtags are ongoing,^25^ limiting our analysis to the hashtag #radonc may fail to capture influential conversations with the larger oncology community that leave out this radiation oncology specific, yet frequently evaluated^1^^,^^26^ hashtag.

Although the degree to which influential Twitter discourse effects patient outcomes remains uncertain, this analysis demonstrates that it is feasible to globally evaluate influential handles and study them over time to understand which entities are influencing the discourse of radiation oncology. In a field as dynamic as radiation oncology, as more users become engaged in conversations on Twitter,^1^ it will be informative to continue evaluating the entities who are influencing the conversation on Twitter, what special interests they may represent, as well as the effects those interests and their influence might have on the specialty at large.

## Appendix Supplementary materials

Image, application 1
