# Supplementary material for: Characterizing Twitter Influencers in Radiation Oncology
Source: Adv Radiat Oncol. 2022 Mar 23;7(6):100919. doi: 10.1016/j.adro.2022.100919 (PMC9184867; doi:10.1016/j.adro.2022.100919)
Supplement: Supplementary file 1 [file mmc1.docx]

**SUPPLEMENTAL MATERIAL**

**Supplemental Table 1: Likelihood Ratio Test of Categorical Variables**

|  | Residual Degrees of freedom | **Residual Sum of Squares** | Degrees of freedom | Sum of Squares | p value |
| --- | --- | --- | --- | --- | --- |
| Category |  |  |  |  |  |
| model w/ category | 89 | 225.89 | NA | NA | NA |
| model w/o category | 94 | 483.79 | -5 | -257.90 | <0.001 |
| Role |  |  |  |  |  |
| model w/ role | 57 | 104.03 | NA | NA | NA |
| model w/o role | 59 | 140.97 | -2 | -36.94 | <0.001 |
| Specialty |  |  |  |  |  |
| model w/ specialty | 44 | 40.68 | NA | NA | NA |
| model w/o specialty | 46 | 58.50 | -2 | -17.82 | <0.001 |
| Practice Type |  |  |  |  |  |
| model w/ practice type | 76 | 212.74 | NA | NA | NA |
| model w/o practice type | 78 | 238.42 | -2 | -25.67 | 0.01 |
| Region |  |  |  |  |  |
| model w/ region | 116 | 249.92 | NA | NA | NA |
| model w/o region | 121 | 366.04 | -5 | -116.11 | <0.001 |
